# G Protein-Coupled Estrogen Receptor-Selective Ligands Modulate Endometrial Tumor Growth

**DOI:** 10.1155/2013/472720

**Published:** 2013-11-27

**Authors:** Whitney K. Petrie, Megan K. Dennis, Chelin Hu, Donghai Dai, Jeffrey B. Arterburn, Harriet O. Smith, Helen J. Hathaway, Eric R. Prossnitz

**Affiliations:** ^1^Department of Cell Biology and Physiology and UNM Cancer Center, The University of New Mexico Health Sciences Center, Albuquerque, NM 87131, USA; ^2^Department of Obstetrics and Gynecology, UNM Cancer Center, The University of New Mexico Health Sciences Center, Albuquerque, NM 87131, USA; ^3^Department of Obstetrics and Gynecology, University of Iowa Carver College of Medicine, Iowa City, IA 52242, USA; ^4^Department of Chemistry and Biochemistry, New Mexico State University, Las Cruces, NM 88003, USA; ^5^Department of Obstetrics and Gynecology and Women's Health, Albert Einstein College of Medicine and Montefiore Medical Center, Bronx, NY 10461, USA

## Abstract

Endometrial carcinoma is the most common cancer of the female reproductive tract. GPER/GPR30 is a 7-transmembrane spanning G protein-coupled receptor that has been identified as the third estrogen receptor, in addition to ER*α* and ER*β*. High GPER expression is predictive of poor survival in endometrial and ovarian cancer, but despite this, the estrogen-mediated signaling pathways and specific estrogen receptors involved in endometrial cancer remain unclear. Here, employing ER*α*-negative Hec50 endometrial cancer cells, we demonstrate that GPER mediates estrogen-stimulated activation of ERK and PI3K via matrix metalloproteinase activation and subsequent transactivation of the EGFR and that ER-targeted therapeutic agents (4-hydroxytamoxifen, ICI182,780/fulvestrant, and Raloxifene), the phytoestrogen genistein, and the “ER*α*-selective” agonist propylpyrazole triol also function as GPER agonists. Furthermore, xenograft tumors of Hec50 cells yield enhanced growth with G-1 and estrogen, the latter being inhibited by GPER-selective pharmacologic antagonism with G36. These results have important implications with respect to the use of putatively ER-selective ligands and particularly for the widespread long-term use of “ER-targeted” therapeutics. Moreover, our findings shed light on the potential mechanisms of SERM/SERD side effects reported in many clinical studies. Finally, our results provide the first demonstration that pharmacological inhibition of GPER activity *in vivo* prevents estrogen-mediated tumor growth.

## 1. Introduction

Carcinoma of the endometrium is the most common cancer of the female reproductive tract with over 40,000 new diagnoses and over 7,000 deaths per year in the United States. Although the majority (~75%) of endometrial tumors are of endometrioid histology (designated type I tumors), expressing high levels of estrogen receptor (ER), progesterone receptor (PR), and epidermal growth factor receptor (EGFR), about 25% of tumors are of advanced stage (designated type II tumors), are unlikely to be ER^+^/PR^+^, and have a poorer prognosis [1]. Although overlap exists with respect to histology, genetic aberations, and epidemiological profiles, the two tumor types appear to represent discrete carcinogenic processes with distinct molecular characteristics. Type I tumors consist of well-differentiated tumors preceded by endometrial hyperplasia and are associated with a loss of PTEN expression as well as abnormalities in *β*-catenin, Kras, and DNA mismatch repair genes. Type 2 tumors are a heterogeneous group of tumors including high-grade (undifferentiated) endometrioid carcinomas, uterine papillary serous carcinomas, clear cell carcinomas, and carcinosarcomas, with different mutational profiles. Over 90% of uterine papillary serous carcinomas are associated with p53 mutations, 45–60% have Her-2/neu mutations, and PTEN mutations are rare [2, 3]. Carcinosarcomas, which are characterized by malignant epithelial and mesenchymal components, are associated with many of the epidemiological risk factors linked to endometrioid carcinomas including obesity and exposure to tamoxifen therapy, suggesting that dysregulated estrogen signaling has a role in their pathogenesis and may represent a therapeutic target. Moreover, recent mutational profiling studies indicate that whereas some carcinosarcomas share mutations with type 1 tumors (PTEN and ARID1A), others share mutations with uterine papillary serous carcinomas (notably p53 and PPP2R1A) [4].

The lack of estrogen receptor *α* (ER*α*) expression in most type II tumors has led to the assumption that these tumors must be “estrogen-independent” and that treatment with antiestrogens (selective estrogen receptor modulators (SERMs) such as tamoxifen and Raloxifene, or pure antagonists/selective estrogen receptor modulators (SERDs) such as ICI182,780/fulvestrant) commonly used in breast cancer treatment, would be ineffectual, a conclusion largely substantiated in a number of clinical trials [5, 6]. In fact, prolonged treatment of breast cancer with SERMs such as tamoxifen leads to an increased incidence of endometrial cancer [7], particularly those of high-risk histologic types [8], resulting in significantly poorer overall survival [9]. The effects of tamoxifen in the uterus have been ascribed to altered expression of nuclear coregulatory proteins in the endometrium compared to the breast, resulting in moderate agonist activity of tamoxifen in the uterus, compared to its antagonistic effects in the breast [10–13]. However, recent results have suggested that a heretofore-underappreciated estrogen receptor, the G protein-coupled estrogen receptor (GPER, formerly GPR30), may play an important role in both the increased incidence of endometrial cancer in women treated with tamoxifen [14] as well as representing an alternate mechanism through which endometrial cancers, particularly type II tumors, can maintain responsiveness to estrogen [15].

GPER is a member of the 7-transmembrane spanning G protein-coupled receptor (GPCR) superfamily, structurally unrelated to the nuclear receptor family members ER*α* and ER*β* [16, 17]. Activation of GPER by estrogen has been demonstrated in many cancer cell lines [18, 19], including endometrial cancer cells [15, 20–27]. GPER is also activated by antiestrogens including tamoxifen (i.e., 4-hydroxytamoxifen) [28] and ICI182,780 (fulvestrant) [29], leading to the suggestion that GPER plays a role in hormone-resistance in breast cancer [30, 31] as well as in the increased incidence of endometrial cancer in women taking tamoxifen for breast cancer [14, 32]. Furthermore, GPER (over)expression has been associated with many cancers and in particular poor prognosis in a number of cancers, including breast [33], ovarian [34], lung [35], pancreatic [36], and endometrial [37] although observations to the contrary have also been reported [38, 39].

Because of the lack of specificity of estrogen and anti-estrogens for the three known estrogen receptors (ER*α*, ER*β*, and GPER), we have developed both a GPER-selective agonist (G-1, [40]) and antagonists (G15 [41] and G36 [42]) that display virtually no activity towards the classical estrogen receptors. In the current study, we examine the expression and function of GPER in the ER*α* ^−^/ER*β* ^−^ endometrial cancer cell line Hec50 [43, 44], which is representative of type II endometrial cancers. We demonstrate that, in Hec50 cells, GPER is localized predominantly in intracellular membranes and mediates PI3K and ERK activation in response to both estrogen and G-1 as well as the antiestrogens tamoxifen, Raloxifene, and ICI182,780 and the “ER*α*-selective” agonist propylpyrazole triol (PPT). We also demonstrate that Hec50 cells and primary patient endometrial adenocarcinomas maintain expression of GPER when grown as xenograft tumors. Finally, we demonstrate both estrogen and G-1 stimulate Hec50 xenograft tumor growth *in vivo* and that the GPER antagonist G36 greatly reduces growth of estrogen-stimulated Hec50 tumors. Overall, these results suggest that GPER may play a critical role in endometrial carcinogenesis, providing a novel target for prognosis and treatment.

## 2. Materials and Methods

*Reagents*. 17*β*-estradiol, 17*α*-estradiol, 4-hydroxytamoxifen, Raloxifene, genistein, LY294002, bovine serum albumin (BSA), normal goat serum, insulin, transferrin, hydrocortisone, fetuin, pancreatin, and trypsin were from Sigma (St. Louis, MO, USA). AG1478 and GM6001 were from Calbiochem (Billerica, MA, USA). DPN, PPT, and ICI182,780 were from Tocris Chemicals (Ellisville, MO, USA). G-1, G15, and G36 were synthesized as previously described [40–42]. Goat anti-rabbit Alexa-488, goat anti-rabbit Alexa-568, and donkey anti-mouse Alexa-568-conjugated secondary antibodies were from Invitrogen (Carlsbad, CA, USA). Rabbit anti-GPER C-terminal antiserum (cross-reactive to both human and murine sequences) was produced as previously described and used at a dilution of 1 : 10,000 [28]. Rabbit anti-GPER human N-terminal antiserum was produced against the peptide sequence MDVTSQARGVGLEMYPGTAQPAAC (with an added carboxy-terminal cysteine for conjugation to KLH) by New England Peptide, Inc. (Gardner, MA, USA) and used at a dilution of 1 : 5000. Polyclonal antibodies against total ERK and pERK were from Cell Signaling (Danvers, MA, USA); monoclonal anti-actin and anti-*β*-catenin antibodies were from Millipore (Burlington, MA, USA). Goat anti-rabbit HRP and donkey anti-mouse HRP were from GE-Amersham (Piscataway, NJ, USA). Dulbecco's MEM, RPMI 1640, and phenol red-free DMEM/F12 media, penicillin/streptomycin/glutamine, and fetal bovine serum were obtained from Fisher (Pittsburgh, PA, USA).

*Cell Culture and Transfection*. Human endometrial carcinoma Ishikawa H cells and Hec50 (specifically Hec50co [44]) cells (kindly provided by K. K. Leslie) were cultured in DMEM medium with FBS (10%) and 100 U/mL penicillin, 100 *μ*g/mL streptomycin, and 2 mm L-glutamine. Cells were grown as a monolayer at 37°C, in a humidified atmosphere of 5% CO_2_ and 95% air. PH-RFP plasmid DNA was transfected with Lipofectamine2000 according to manufacturer's directions but using 1/4 the recommended amount of DNA. For “co-transfection” of PH-RFP plasmid DNA and siRNA on coverslips, Lipofectamine2000 was used to transfect siRNA on day 1 according to manufacturer's directions; on day 2, PH-RFP plasmid with additional siRNA as on day 1 was retransfected. For GPER knockdown, siRNA targeting GPER (ON-TARGET plus SMARTpool L-005563-00) was obtained from Dharmacon/Thermo-Fisher (Lafayette, CO, USA). The nontargeting siRNA ON-TARGETplus siControl Non-Targeting siRNA (D-001810-02) was used as a control. Cells transfected with siRNA were used in PH assays and stained for GPER expression 48 hours following the second siRNA transfection. For microscopy experiments, cells were seeded onto 12 mm glass coverslips and allowed to adhere for at least 24 h prior to antibody staining or 12 h prior to transfection.

Primary mouse uterine epithelial cells were harvested and cultured as described [45]. Briefly, uteri were removed from C57Bl6 mice between 21 and 35 days of age and slit longitudinally, followed by incubation in 2.5% pancreatin and 0.5% trypsin for 1 h at 4°C, followed by 1 h at RT. Digested uteri were then briefly vortexed, releasing epithelial sheets and fragments, which were transferred to a fresh tube containing 2% trypsin inhibitor (Invitrogen) in Hanks Balanced Salt Solution (HBSS). After two washes with HBSS, cells were seeded directly onto acid-washed, poly-L-lysine-coated glass coverslips and cultured in serum-free DMEM/F12 medium supplemented with 5 *μ*g/mL insulin, 10 *μ*g/mL transferrin, 10^−7^ M hydrocortisone, 2 mg/mL BSA, 1 mg/mL fetuin, and antibiotics.

*Immunofluorescence Staining*. Cells were seeded on 12 mm glass coverslips and fixed with 4% PFA (Electron Microscopy Sciences, Hatfield, PA, USA) in PBS for 15 min at 37°C. Coverslips were washed three times with PBS and blocked for 1 h with 3% BSA in PBS. Where indicated, permeabilization was accomplished with 0.05% Triton X-100 in the blocking buffer. Primary antibody was diluted in 3% normal goat serum and coverslips were incubated for 4 h at room temperature. Coverslips were washed three times with PBS and incubated with secondary antibody diluted in 3% normal goat serum. Coverslips were washed three times with PBS and mounted with Vectashield containing DAPI (Vector Labs, Burlingame, CA, USA). Confocal fluorescence images were collected on a Zeiss LSM 510 confocal microscope. Typical cell lengths ranged from 20 to 35 *μ*m.

*Western Blotting*. Cells were harvested directly for receptor expression or starved in phenol red-free RPMI 1640 for 24 h prior to treatment. Cells were washed once with ice-cold PBS and lysed using NP-40 buffer. Twenty *μ*g protein was loaded per lane and electrophoresed on 4–20% SDS-PAGE gels (Thermo Scientific, Waltham, MA, USA), transferred to PVDF membrane (Millipore), and blocked with 3% BSA in TBST (50 mm Tris, 150 mm NaCl, and 0.1% Tween-20) before overnight incubation with primary antibodies at 4°C. Blots were incubated with HRP-conjugated secondary antibodies, developed using SuperSignal West Pico Chemiluminescent Substrate (Thermo Scientific), exposed to film, scanned, and quantified using Image J software (NIH). 

*PI3K Activation*. The pleckstrin homology (PH) domain (responsible for PIP3 binding) of Akt [46] fused to monomeric red fluorescent protein 1 (mRFP1) [47] generated the PH-RFP construct, which was used to localize sites of increased cellular PIP3 accumulation [28]. Hec50 cells were plated on coverslips, transfected with PH-RFP, followed by a 24 h recovery in complete DMEM, and serum starved in phenol red-free DMEM/F12 for 24 h before stimulation with ligands as indicated. The cells were fixed with 2% PFA in PBS, washed, mounted in Vectashield containing DAPI (Vector Labs), and imaged by confocal microscopy using a Zeiss LSM510 confocal fluorescence microscope. Images are representative of 75–85% of the transfected cells observed. 

*Xenograft Tumors*. Ishikawa H cell and Hec50 cell xenograft tumors were produced by injecting ~3 × 10^6^ cells (in 100 *μ*L DMEM) subcutaneously into 6–8-week-old female athymic, Crl:Nu/Nu-nuBR “athymic nude” mice [48]. Subcutaneous tumors were recovered for histology and immunohistochemistry typically ~6 weeks after injection, when the tumors reached ~10 mm in diameter. For treatment models, Hec50 cells were injected into ovariectomized athymic nude mice 10 days after ovariectomy. Individual 60-day-release sham, estrogen (1.5 mg), G-1 (2.25 mg (equimolar with estrogen)), and G36 (11 mg pellets, (5-fold molar excess versus estrogen and G-1)), custom made by Innovative Research of America (Sarasota, FL), were introduced subcutaneously near the scapula with a trochar on the same day as Hec50 cell inoculation. When tumors became palpable (3–5 mm in diameter), tumor size was measured ~3 times per week by digital caliper, and upon sacrifice tumors were dissected and weighed. All protocols were approved by the Institutional Animal Care and Use Committee of the University of New Mexico Health Sciences Center.

For xenografts of fresh human tumor samples, tumors were obtained from the Department of Pathology at the University of New Mexico immediately after their arrival at the Surgical Pathology Gross Room. Fat and necrotic tissues were trimmed and remaining tumor tissues were rinsed with cell culture medium (DMEM). Tumors were minced into a fine homogenate and mixed with medium. Typically, 10 mg of tumor tissue was mixed with 100 uL medium for subcutaneous injection into a 6–8-week old athymic Crl:Nu/Nu-nuBR female mouse to create the first-generation xenografts, which were used for analysis as reported previously [49]. The collection of human endometrial tumors was approved by Human Research Protections Office at the University of New Mexico. 

*Histological Staining of Tumors*. Five-micron sections from paraffin-embedded tumor tissues were prepared for immunohistochemistry (IHC) as previously described using the carboxy-terminus-targeted antibody against GPER [37]. In brief, sections were deparaffinized in CitriSolv clearing agent (Fisher, Pittsburgh, PA, USA) followed by rehydration in increasing H_2_O : ethanol solutions. Antigen retrieval was accomplished by microwaving slides in 0.01 M sodium citrate buffer (pH 6.0) for 25 min, followed by incubation of cooled slides in fresh 2% H_2_O_2_ for 10 min. Permeabilization and blocking were performed by incubating the slides for 30 min in 200 *μ*L of 0.1% Triton X-100 in PBS with 3% bovine serum albumin in a humid chamber. Slides were incubated with the affinity-purified GPER carboxy-terminal antibody diluted to a final protein concentration of 2 *μ*g/mL in 3% normal goat serum for 1 h. Following multiple washes, bound antibody was detected using the immunoperoxidase system by incubating with goat anti-rabbit IgG conjugated to horseradish peroxidase (diluted 1 : 250 in 3% normal goat serum) for 45 min. Peroxidase was detected with the enzyme substrate 3′,3-diaminobenzidine tetrahydrochloride (DAB; Sigma, St. Louis, MO, USA).

## 3. Results

### 3.1. GPER Is Expressed Intracellularly in Hec50 Type II Endometrial Cancer Cells

**Figure 1:**
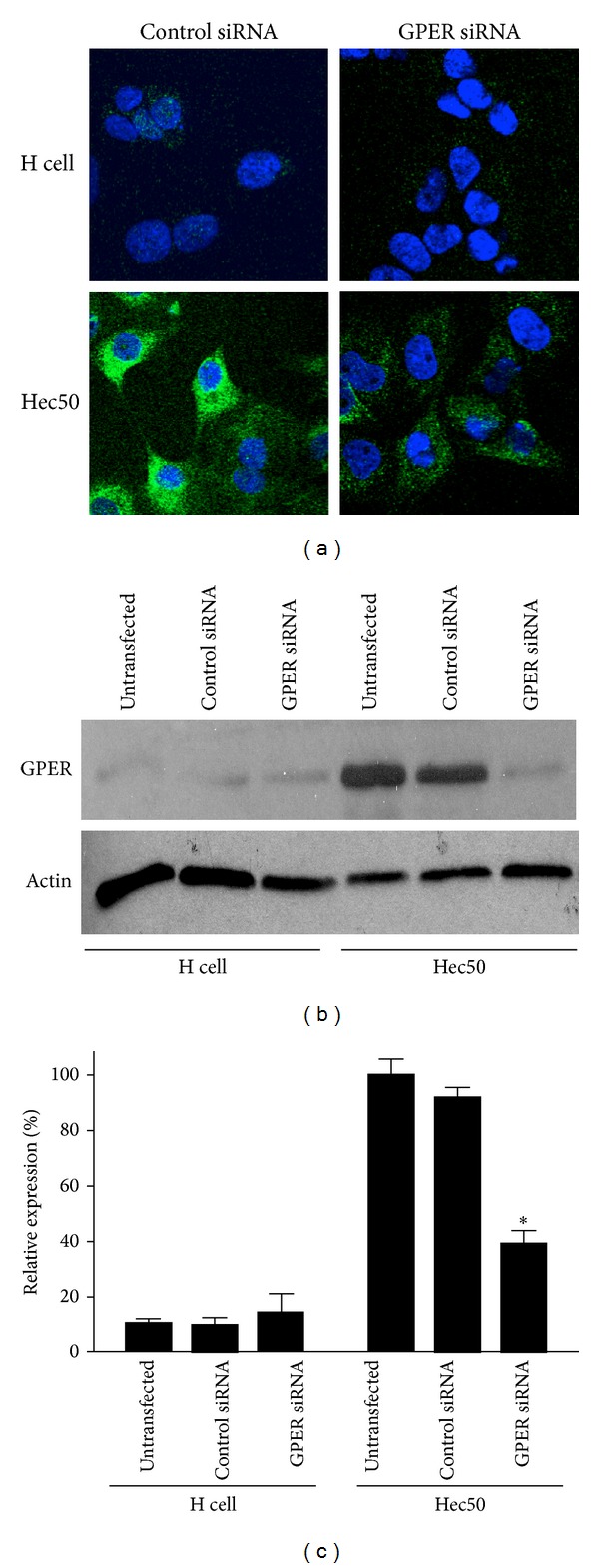
GPER expression in type I and type II endometrial cancer cells. (a) Representative immunofluorescence images of Ishikawa H cells (type I) and Hec50 cells (type II) transfected with either control siRNA or siRNA targeting GPER. GPER is shown in green; nuclei are stained blue with DAPI. (b) Western blot of GPER and actin in untransfected, control siRNA- and GPER-targeted siRNA-transfected H cells and Hec50 cells. (c) Western blot quantitation of GPER expression relative to actin and normalized to untransfected Hec50 cells. Data represent mean ± s.e.m. from three experiments. *P < 0.05 compared to control siRNA.

Although the physiological and biological effects of estrogen have traditionally been described as being mediated by the nuclear estrogen receptors ER*α* and ER*β*, recent evidence suggests an increasing role for the 7-transmembrane estrogen receptor GPER [16]. We have previously observed that, in many cell types, staining for GPER reveals a predominantly intracellular pattern associated with the endoplasmic reticulum and Golgi apparatus. Intracellular localization, including the nucleus [50, 51], has been reported by many [52–55] but not in other studies [56–58]. Recent results suggest that GPER undergoes constitutive internalization, which would suggest that at steady state a preponderance of GPER would be detected as intracellular [59, 60]. To address this further in endometrial epithelial cancer cells, we examined expression in type II Hec50 cells and compared it to type I Ishikawa H cells. Immunofluorescence staining and Western blotting (both with an anti-GPER carboxy terminus-targeted antibody) revealed that Ishikawa H cells express very low levels of GPER (~10%) compared to Hec 50 cells (Figures 1(a) and 1(b)). Treatment of both cell types with siRNA resulted in a significant reduction of GPER expression in Hec50 cells with no significant reduction in H cells (Figure 1). Immunofluorescence staining also revealed a pattern of GPER staining throughout the cytoplasm, consistent with localization to intracellular membranes.

**Figure 2:**
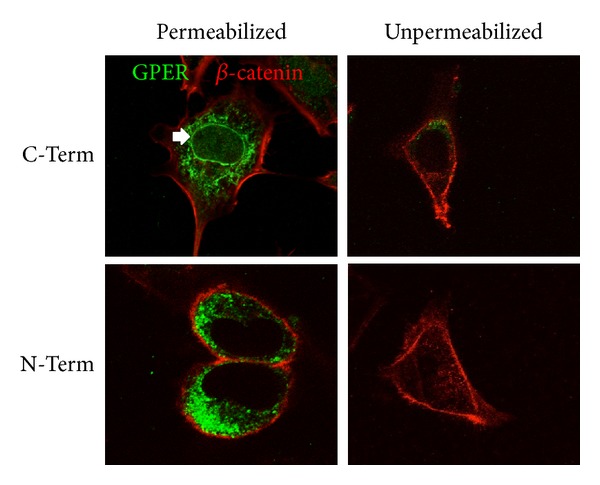
Subcellular localization of GPER expression in primary mouse uterine epithelial cells. Cells were stained with either an antibody targeted against the carboxy terminus (C-Term) or amino terminus (N-Term) of GPER under either permeabilizing or nonpermeabilizing conditions. As the amino terminus of GPCRs would be extracellular, any plasma membrane-localized receptor should be stained by the amino terminus-targeted antibody under nonpermeabilizing conditions. GPER is stained green; *β*-catenin is stained red as a plasma membrane marker. Arrow indicates nuclear membrane.

To assess the localization of GPER in greater detail in primary cells, we cultured freshly isolated mouse uterine epithelial cells on glass coverslips and stained for both the carboxy-terminus as well as the amino-terminus of GPER in combination with *β*-catenin as a cell surface marker under both permeabilizing and nonpermeabilizing conditions (Figure 2). Staining for GPER with both antibodies under permeabilizing conditions revealed an intracellular localization with no significant overlap with *β*-catenin. Interestingly, in many cells (e.g., upper left panel), there was also staining of the nuclear membrane, which is continuous with the endoplasmic reticulum. Since GPCRs are oriented in the plasma membrane with their amino terminus to the cell exterior and their carboxy terminus to the cell cytoplasm, we expected that if GPERs were expressed in the plasma membrane, staining with an antibody targeting the amino terminus should be able to detect any receptor in the plasma membrane in the absence of permeabilization. However, staining with the amino terminus-targeted antibody in the absence of permeabilization revealed no significant staining, in contrast to the staining observed following permeabilization, indicating that little GPER is expressed on the cell surface compared to the intracellular pool.

### 3.2. PI3K Activation by Estrogen in Hec50 Endometrial Cancer Cells Is Mediated by GPER

As Hec50 cells lack ER*α* expression [44], we next asked whether, in the absence of ER*α*, estrogen could still mediate rapid signaling. To address this, we utilized a method we have previously employed [28, 41, 42], monitoring the activation of PI3K through the translocation of a fluorescent reporter of PIP3 localization, namely, a red fluorescent reporter (mRFP1 [47]) protein fused to the PH (PIP3-binding) domain of Akt [46]. In previous studies, we have observed that in serum-starved unstimulated cells (e.g., COS7 cells), the PH-RFP reporter is fairly uniformly distributed throughout the cytoplasm and nucleus [28]. However, when expressed in certain cancer cell lines (e.g., SKBr3 breast cancer cells), the reporter exhibits an enhanced plasma membrane localization [28], even under serum-starved conditions, that is likely due to constitutive activation of signaling pathways (e.g., EGFR activation or Her2 overexpression) that lead to activation of PI3K in the absence of exogenous stimuli.

**Figure 3:**
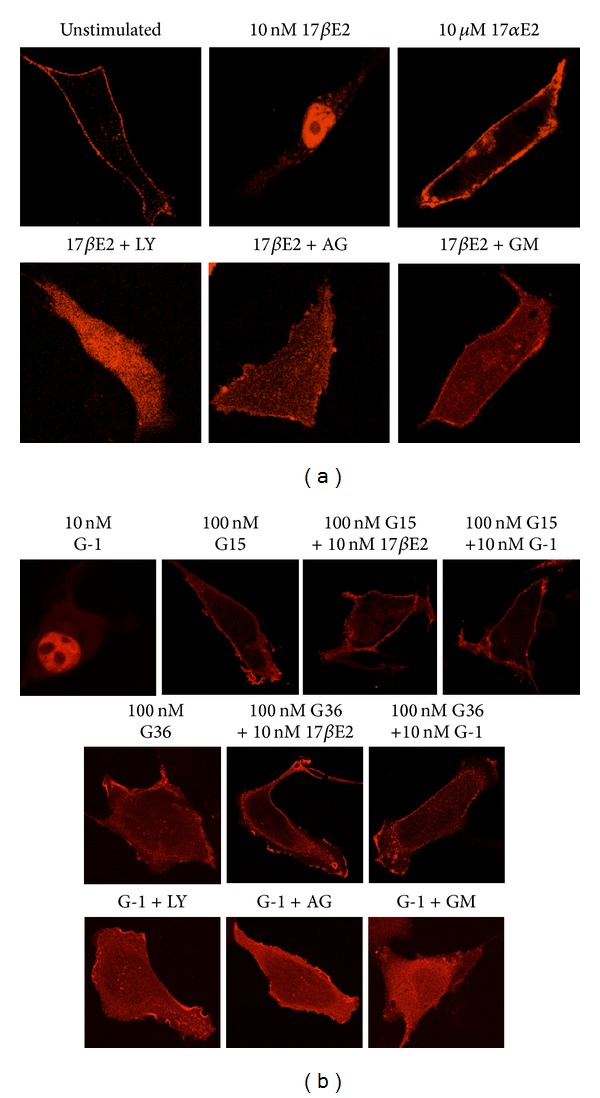
Activation of PI3K by estrogen in Hec50 cells is mediated by GPER. Hec50 cells were transfected with a marker of PIP3 production, the PH domain of Akt fused to monomeric red fluorescent protein (mRFP) yielding the marker PH-RFP. (a) PH-RFP-transfected Hec50 cells were stimulated with the following ligands: 10 nM estrogen (17*β*E2), 10 *μ*M 17*α*-estrogen (17*α*E2), or 10 nM estrogen in the presence of the PI3K inhibitor LY294001 (10 *μ*M, 20 min pre-incubation; 17*β*E2 + LY), the EGFR inhibitor AG1478 (25 *μ*M, 60 min pre-incubation; 17*β*E2  +  AG) or the metalloproteinase inhibitor GM6001 (10 *μ*M, 30 min pre-incubation; 17*β*E2 + GM). Unstimulated designates vehicle only. (b) PH-RFP-transfected Hec50 cells were stimulated for 15 min with the GPER-selective agonist G-1 or estrogen at the indicated concentrations in the absence or presence of the GPER-selective antagonists G15 and G36 (cells were pretreated 15 min with G15 or G36 prior to stimulation with E2 or G-1) or in the presence of 10 nM G-1 and the PI3K, EGFR or metalloproteinase inhibitors as in (a).

To determine whether estrogen mediates rapid activation of PI3K in Hec50 cells, we transfected cells with PH-RFP and subsequently treated serum-starved cells with estrogen (17*β*-estradiol). The unstimulated cells yielded a plasma membrane localization similar to that previously observed in SKBr3 breast cancer cells, suggesting constitutive activation of PI3K at the plasma membrane (Figure 3(a)). However, upon estrogen stimulation for 15 min, the reporter translocated to the nucleus, suggesting activation of PI3K in the nucleus, as has been suggested by studies characterizing a nuclear pool of PI3K [61, 62]. Importantly, the inactive stereoisomer of estrogen (17*α*-estradiol) did not demonstrate PI3K activation, even at 1000x the concentration of 17*β*-estradiol, demonstrating the stereoselectivity of the receptor involved for the physiologically active isomer of estrogen. To test whether the activity of PI3K was required for the translocation of the PH-RFP reporter, we pretreated cells with the PI3K inhibitor LY294002, followed by estrogen stimulation, which yielded a uniform distribution of the PH-RFP reporter throughout the cell. This indicated not only that the nuclear localization of the PH-RFP reporter required PI3K activity, but that the membrane localization in unstimulated cells was also due to PI3K activity (as LY294002 treatment in the absence of estrogen yielded an identical distribution, data not shown). As rapid estrogen signaling has been demonstrated to involve/require EGFR activation through the generation of HB-EGF [29], we also pretreated cells with the EGFR kinase inhibitor AG1478 and the metalloproteinase inhibitor GM6001 (to block HB-EGF production). Both inhibitors blocked estrogen-mediated nuclear accumulation of the PH-RFP reporter, indicating a requirement for both HB-EGF and EGFR in estrogen-mediated PI3K activation in Hec50 endometrial cancer cells.

As Hec50 cells lack expression of the classical estrogen receptor ER*α* but express GPER, we next examined whether the activation of PI3K by estrogen might be mediated by GPER. Using the GPER-selective agonist G-1, we observed that, like estrogen, the PH-RFP reporter translocated to the nucleus, suggesting estrogen might be mediating its effects via GPER (Figure 3(b)). In support of this, the GPER-selective antagonists G15 and G36 not only prevented G-1-mediated activation of PI3K but also blocked estrogen-mediated PI3K activation (Figure 3(b)). G15 and G36 alone had no effect. As observed for estrogen-mediated activation of GPER, PI3K activation in response to G-1 also requires both EGFR kinase and metalloproteinase activity, as AG1478 and GM6001 also blocked nuclear translocation of PH-RFP following G-1 stimulation.

**Figure 4:**
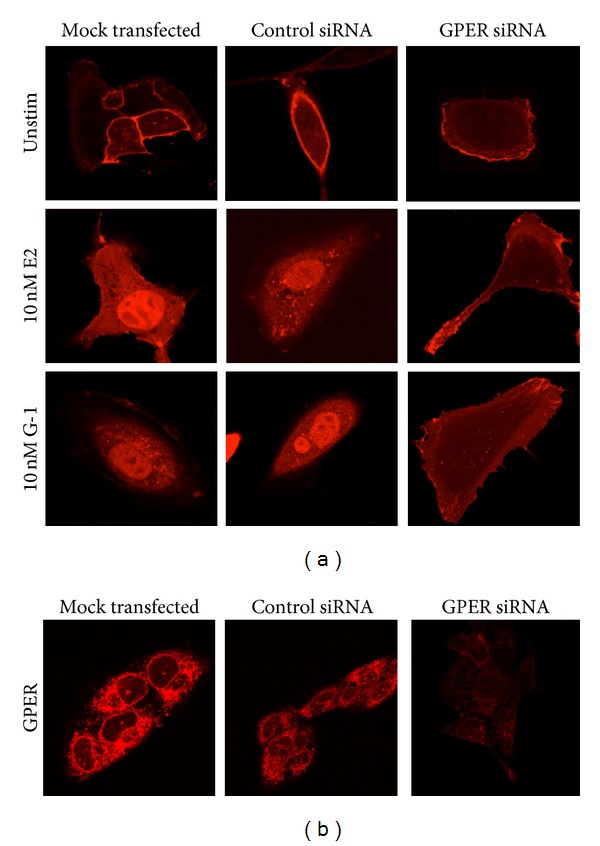
GPER mediates PI3K activation in Hec50 cells. (a) Hec50 cells were transfected with no siRNA (mock transfected), control siRNA or siRNA targeting GPER (GPER siRNA), and the PH-RFP reporter. Transfected Hec50 cells were stimulated with vehicle, estrogen (10 nM E2), or G-1 (10 nM). (b) Hec50 cells transfected with no siRNA (mock transfected) and control siRNA or siRNA targeting GPER (GPER siRNA) were stained for GPER (with carboxy-terminal antibody) to demonstrate the specific knockdown of GPER in the GPER siRNA-treated cells.

To further demonstrate the requirement for GPER in PI3K activation by estrogen and G-1 beyond pharmacological inhibition, we employed siRNA to knockdown expression of GPER (Figure 4). In mock-transfected (no siRNA) and control siRNA-transfected Hec50 cells, both estrogen and G-1 stimulated nuclear localization of the PH-RFP reporter. However, in cells transfected with GPER-targeted siRNA, neither estrogen nor G-1 stimulation resulted in nuclear translocation of the PH-RFP reporter (Figure 4(a)). Knockdown of GPER protein was confirmed by immunofluorescence staining of mock, control, and GPER siRNA-transfected cells (Figure 4(b)). The use of both a pharmacological approach (G15 and G36) and siRNA to prevent activation of PI3K by estrogen, as well as the ability of G-1 to activate PI3K, strongly indicates that GPER is the receptor mediating responsiveness to estrogen in Hec50 cells.

### 3.3. Multiple Estrogen Mimetics Activate PI3K and ERK via GPER

**Figure 5:**
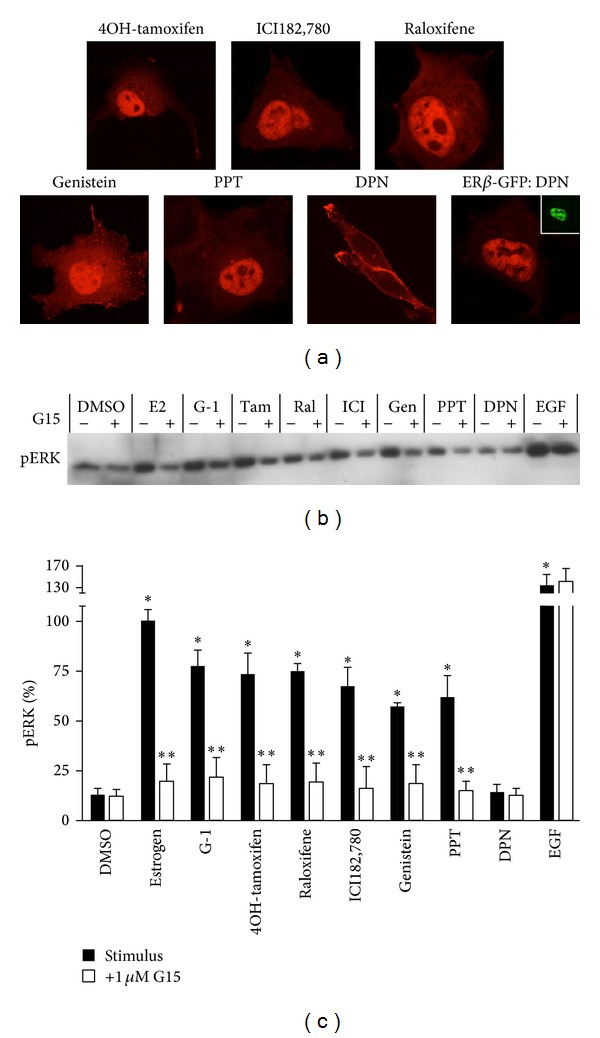
GPER-mediated activation of PI3K and ERK in Hec50 cells by SERMs, a SERD, and an ER*α*-selective agonist. (a) PH-RFP-transfected Hec50 cells were stimulated for 15 minutes with the following ligands: 4-hydroxytamoxifen (100 nM, 4OH-tamoxifen), ICI182,780 (100 nM), Raloxifene (100 nM), genistein (100 nM), PPT (100 nM), or DPN (10 *μ*M). Hec50 cells were also cotransfected with ER*β*-GFP (shown in inset) and PH-RFP and stimulated with 100 nM DPN to verify the activity of DPN as an ER*β* agonist in Hec50 cells (ER*β*-GFP:DPN). (b) Hec50 cells were stimulated for 15 min with vehicle (0.05% DMSO), estrogen (10 nM, E2), G-1 (10 nM), 4-hydroxytamoxifen (100 nM, Tam), Raloxifene (100 nM, Ral), ICI182,780 (100 nM, ICI), genistein (100 nM, Gen), PPT (100 nM), DPN (10 *μ*M), or EGF (1 nM) either in the presence or absence of 1 *μ*M G15 (10 min pretreatment, with 0.05% DMSO in samples without G15). (c) Band intensities of pERK were normalized to total ERK and plotted with estrogen as 100%. Data represent mean ± s.e.m. from three experiments. *P < 0.05 versus DMSO; **P < 0.05 versus paired stimulus without G15.

To examine the effects of a number of therapeutic antiestrogens and other ligands on PI3K activation in ER*α* ^−^/*β* ^−^ Hec50 cells, we evaluated PH-RFP localization in cells treated with 4-hydroxytamoxifen, ICI182,780, the benzothiophene-based and recently FDA-approved SERM Raloxifene, the phytoestrogen genistein, and the widely used ER*α*- and ER*β*-selective agonists propylpyrazoletriol (PPT) and diarylpropionitrile (DPN) (Figure 5(a)). There is evidence that ICI182,780 [29] and tamoxifen [15, 26, 28] can act through GPER to stimulate rapid cellular signaling. Specifically, we have demonstrated tamoxifen-mediated stimulation of PI3K in GPER-transfected COS7 cells and GPER^**+**^ SKBr3 breast cancer cells (both of which do not express ER*α* or for that matter ER*β*) [28]. PPT and DPN have been used extensively as “selective” agonists of ER*α* and ER*β*, respectively [63, 64]. Whereas PPT displays ~400-fold binding selectivity for ER*α* over ER*β*, DPN exhibits only ~70-fold selectivity for ER*β* over ER*α* [65–68]. Of these, all compounds (at 100 nM), with the exception of DPN (even at 10 *μ*M), stimulated the nuclear translocation of the PH-RFP reporter to the nucleus (Figure 5(a)), as observed with estrogen and G-1 (Figures 3 and 4). To confirm the activity of DPN, we cotransfected Hec50 cells with ER*β*-GFP and PH-RFP. In cells expressing ER*β*, DPN was indeed able to stimulate PH-mRFP translocation at a concentration of 100 nM, demonstrating the ability of Hec50 cells to respond to DPN via ER*β* and furthermore demonstrating that, without the exogenous expression of ER*β*, Hec50 cells do not express sufficient ER*β* (if any) to respond to DPN.

We and others have previously demonstrated that GPER is capable of activating ERK in addition to PI3K in multiple cancer and other cell lines, including other endometrial cancer cell lines [22, 27, 42, 69]. To further examine and quantify the ligand specificity of GPER, we determined pERK levels in Hec50 cells stimulated with the ligands above (Figures 5(b) and 5(c)). Although the only known estrogen receptor expressed in Hec50 cells is GPER, we confirmed the contribution of GPER to ligand-induced ERK activation using the GPER-selective antagonist G15. As for PI3K activation, estrogen, G-1, 4-hydroxytamoxifen, ICI182,780, and Raloxifene stimulated pERK ~5–8-fold. Activation of ERK by each of these ligands was completely inhibited by G15, indicating an essential role for GPER in the response to each ligand. The phytoestrogen genistein also acted as an agonist of GPER-mediated ERK activation in Hec50 cells and as observed with PI3K activation, PPT (100 nM), but not DPN (at concentrations up to 10 *μ*M), was able to induce ERK activation in a GPER-dependent (i.e., G15-sensitive) manner. Finally, to ensure that G15 did not inhibit ERK activation downstream of the transactivated EGFR, we directly stimulated Hec50 cells with EGF. EGF was ~30% more potent than the next most potent ligand, estrogen, but unlike the other agonists of ERK activation via GPER, EGF stimulation was unaffected by G15, demonstrating that the inhibitory action of G15 on GPER activation is upstream of EGFR.

### 3.4. Expression of GPER in Xenografts of Endometrial Cancer Cell Lines and Human Endometrial Cancers

Hec50 cells are poorly differentiated endometrial cancer cells that were originally isolated from a metastatic lesion in a patient with advanced endometrial cancer who ultimately succumbed to the cancer [70, 71]. The cells do not form glands in tissue culture or in xenografts and do not express either ER*α* or PR [43]. They do however exhibit the capacity to subdifferentiate into a papillary serous phenotype when injected intraperitoneally in mice [72]. Thus, Hec50 cells are an excellent model of type II endometrial tumors [71]. In contrast, Ishikawa H cells were derived from a patient with stage 2 moderately differentiated endometrial adenocarcinoma who was treated with surgery and chemotherapy and survived without recurrence. These cells produce mucous, contain vacuoles, express both ER*α* and PR, and are thus an excellent model of type I endometrial cancer [71].

**Figure 6:**
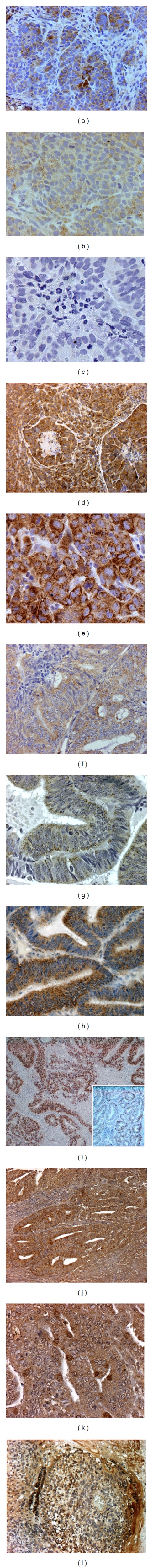
GPER expression in xenografts of Ishikawa H cells and Hec50 endometrial cancer cells as well as representative type I and type II human primary tumors. (a)–(e) Immunohistochemical staining of GPER in xenograft tumors of Ishikawa H cells ((a), 20x; (b), 40x) demonstrating the areas most strongly positive for GPER, and representative xenograft tumors of Hec50 cells ((d), 20x; (e), 40x; (c), negative control (irrelevant primary antibody), 40x). ((f)–(h)) GPER staining of a recurrent adenocarcinoma with endometrioid features ((f), mouse xenograft of patient tumor, 40x; (g), patient tumor, 40x; (h), patient tumor, 20x, demonstrating focal positivity; (i), ER*α* and PR (inset) staining of the same patient tumor). ((j)–(l)) GPER staining from a patient with Stage IA carcinosarcoma ((j), mouse xenograft of patient tumor, 20x, illustrating diffuse positive staining in both epithelial and stromal fractions; (k), patient tumor, 40x; (l), patient tumor, 20x, demonstrating similarly strong GPER staining in both the epithelial and stromal compartments).

As demonstrated in Figure 1 by immunofluorescence and Western blotting, Hec50 cells express substantially (≥10-fold) more GPER than do H cells. In xenografts, H cells form endometrioid tumors whereas Hec50 can differentiate into a serous subtype [44]. To assess whether GPER expression patterns are maintained in xenografts, we performed immunohistochemistry for GPER on xenograft tumors of both H cells and Hec50 cells from nude mice (Figure 6). Tumors derived from H cells exhibited well-differentiated gland formation (Figures 6(a) and 6(b)), whereas, in tumors derived from Hec50 cells, cells were poorly or undifferentiated, nuclei were pleomorphic, and mitotic activity was abundant (Figures 6(d) and 6(e)). In addition, although GPER was detected to varying extents in the tumors from H cells (Figures 6(a) and 6(b)), it was expressed at far greater levels in tumors from Hec50 cells (Figures 6(d) and 6(e)), consistent with expression observed in cultured monolayers (Figure 1) [28].

We have previously reported that, in endometrial cancer, high GPER expression is prognostic of poor survival [37]. Moreover, in carcinosarcoma subtypes, advanced stage disease was more frequently associated with high levels of GPER and ER*β* expression [73]. To assess whether GPER expression levels and patterns are maintained in xenografts of primary patient tumors, xenografts from tumors preoperatively characterized as type I and type II tumors were generated and immunostained for GPER using xenograft tissues and parallel paraffin-embedded tissue from the original patient tumor (Figures 6(f)–6(l)). Case studies of our illustrated cases are relevant. Patient 1 was originally diagnosed with superficially invasive (24%) grade 1, ER*α* ^+^/PR^+^, endometrioid adenocarcinoma (FIGO stage IB) and presented two years later at our institution with a 20 × 21 × 10 cm mass involving the omentum, anterior abdominal wall, and bowel, which was resected. The patient refused postoperative chemotherapy and was instead treated with tamoxifen 40 mg daily and medroxyprogesterone acetate 200 mg daily, cycle day 16–30, on an IRB-approved institutional trial. With 6 months of hormonal therapy, the patient has remained radiographically free of disease (duration of followup: 4.5 years). Immunostaining of the recurrent tumor (the primary was unavailable for comparison) was scored as PR (3+, 10% of viable epithelial cells), ER*α* (3+, 100% of viable cells), and GPER (3+, less than 10% of viable cells, Figures 6(f)–6(i)).

Patient 2 was diagnosed with grade 3 adenocarcinoma based upon endometrial biopsy but at surgery was found to have carcinosarcoma, FIGO stage IA, with high-grade sarcomatous features. Following weekly cisplatin chemotherapy and radiation therapy this patient has remained disease-free 4 years after therapy. As carcinosarcoma tumors are considered biphasic (i.e., defined by having malignant epithelial and stromal compartments) [37, 74], we evaluated both epithelial and stromal cell staining for GPER expression and observed that both components were strongly positive for GPER. These results demonstrate not only that Hec50 cells maintain high levels of GPER expression as xenografted tumors, but also that Hec50 cells mimic the levels of GPER expression observed in high grade endometrial tumors, as detected in paraffin-embedded patient samples and xenografted patient tumors, suggesting that primary xenografts of endometrial cancers (even complex tumors with biphasic characteristics) may represent an excellent model to test the therapeutic efficacy of GPER-targeted therapies.

Interestingly, within our endometrial cancer repository, we identified six patients who received tamoxifen-based therapy for recurrent endometrial cancer, and of these only one patient (case 1, above, with low GPER expression) experienced a complete response; in contrast, all nonresponders displayed increased GPER expression (defined as expression above the mean [37]) by immunohistochemistry of paraffin-embedded tumor samples (Fisher's *P* = 0.03), suggesting that a lack of or low GPER expression may be a predictor of tamoxifen responsiveness in endometrial cancer.

### 3.5. GPER Antagonist Inhibits Endometrial Tumor Growth

**Figure 7:**
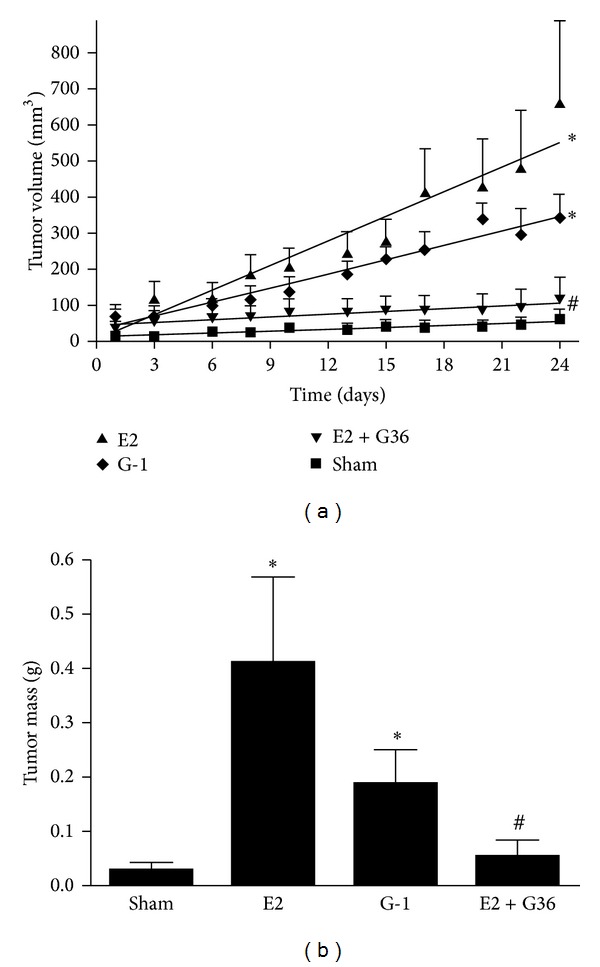
Inhibition of Hec50 tumor growth by GPER antagonist. Hec50 cell tumors were initiated in ovariectomized athymic mice and treated with a slow release pellet containing either no added compound (sham), estrogen (E2), G-1, or with two pellets (one of estrogen and one of G36, E2 + G36). (a) Tumor volume was measured with calipers over a 24-day period. (b) Upon sacrifice, tumors were dissected and tumor mass was determined. Data represent mean ± s.e.m. from 6–8 mice. *P < 0.05 versus sham; ^#^ *P* < 0.05 versus E2 alone. Note that the tumor size for E2 + G36 was not significantly different from the sham.

Type I endometrial cancer can result from excess and/or unopposed estrogen use and typically progresses from hyperplasia to atypical hyperplasia and finally to carcinoma. Type I tumors commonly express ER and PR [75] and are generally responsive to hormone treatment with therapeutic efficacy positively correlating with the level of receptor expression [2, 75, 76]. However, type II endometrial cancers are believed to develop through molecular pathways involving p53 mutations, which more closely resemble high-grade serous ovarian tumors in molecular alterations and morphology and commonly do not express either ER or PR [2, 44], suggesting that estrogen (or its inhibition) should have no effect on the growth of such tumors. To examine the estrogen dependence of a type II endometrial cancer *in vivo*, we generated Hec50 cell xenografts in mice. To determine the effect of estrogen, the tumors were initiated in ovariectomized mice, and estrogen was restored using slow release pellets (Figure 7). Surprisingly, we found very little tumor growth in ovariectomized mice (compared to our experience in ovary intact mice, data not shown). However, upon supplementation with estrogen, tumor growth was increased about 10-fold (compared to ovariectomized/sham-treated mice). To determine whether GPER was responsible for this estrogen-mediated enhancement of tumor growth, we also treated ovariectomized mice with slow release pellets containing G-1. The tumors in these mice were about 5-fold larger than those in sham-treated mice, suggesting that GPER was indeed capable of stimulating tumor growth *in vivo*. Finally, to further test whether the estrogen-stimulated tumor growth was mediated by GPER, we cotreated estrogen-supplemented mice with our recently identified GPER-selective antagonist G36 [42]. Estrogen-stimulated tumor growth was reduced by G36 to that of the sham treatment (i.e., estrogen deprived state), demonstrating a critical role for GPER in the estrogen-mediated response of ER^−^ type II endometrial cancer growth.

## 4. Discussion

Endometrial cancers, like most cancers, consist of multiple, distinguishable tumor types. At a minimum, endometrial cancers have been separated into type I and type II endometrial cancers, based on morphology and molecular phenotypes/genotypes [2]. Recent genomic studies from The Cancer Genome Atlas are shedding more light on the extent and range of mutations associated with this cancer, defining 4 categories of endometrial cancer [77]. Whereas survival is high among women with type I endometrial cancers, the opposite is true for type II cancers, which express high levels of GPER [37]. We have therefore focused our investigations on a model of type II endometrial cancer in this study, particularly due to the fact that these cancers are typically described as estrogen unresponsive due to their lack of ER expression. In this work, we have demonstrated that Hec50 cells, typical of type II endometrial cancer cells that do not express the classical ER*α*, do express GPER, which makes them responsive to estrogen in terms of rapid cellular signaling (PI3K and ERK). Furthermore, as chronic tamoxifen use (i.e., for breast cancer) results in an increased incidence of endometrial cancer, we demonstrated that, in endometrial cancer cells, GPER mediates cellular signaling in response to two SERMs (tamoxifen and Raloxifene) as well as a SERD (ICI182,780), revealing a possible additional mechanism for the increased risk of endometrial cancer with tamoxifen use. We also demonstrated that the ER*α*-selective (i.e., selective versus ER*β*) agonist PPT activates GPER, raising questions about the conclusions drawn from its use in defining exclusive ER*α* function in a multitude of biological systems. Finally, we established that GPER is highly expressed in type II tumors, that Hec50 xenograft tumors display a strong dependence on estrogen *in vivo*, and that this occurs through GPER, the inhibition of which blocks estrogen-stimulated tumor growth.

Although GPCRs are traditionally thought of as cell surface receptors, mediating transmembrane signaling of membrane-impermeable ligands (e.g., ionic small molecules, peptides, and proteins), we originally described GPER localization as being predominantly intracellular [28]. Those and our continuing results have localized GPER primarily to intracellular membrane compartments (endoplasmic reticulum and Golgi membranes), even using immunohistochemistry of tumor and other tissues [34, 37, 53, 54]. In Hec50 cells, we again observed a strong intracellular localization. To examine whether this localization was also evident in primary cells (as opposed to cancer cell lines), we isolated and stained primary murine uterine epithelial cells. As with Hec50 cells, the staining pattern of GPER appeared intracellular but with a more punctate morphology. Consistent with an intracellular localization, no staining was observed using an antibody targeting the amino terminus of GPER under nonpermeabilizing conditions. In a fraction of cells, staining of the nuclear membrane was evident. As the endoplasmic reticulum is continuous with the nuclear membrane, such a pattern would not be unexpected. However, since nuclear membrane staining is not present in all cells and cell types, GPER localization to the nuclear and other membranes may be actively regulated. In fact, others have reported plasma membrane localization to varying extents in diverse cell types [56–58]. Whether the localization and trafficking are dynamically regulated within a cell, or simply different in distinct cell types, remains to be determined. The lack of extensive plasma membrane localization under steady-state conditions suggests either that GPER traffics poorly to the plasma membrane (being retained in the endoplasmic reticulum and Golgi apparatus) or that, if trafficked to the plasma membrane, it is rapidly internalized. Evidence for both of these mechanisms has been recently presented with Filardo et al. revealing constitutive internalization of GPER to a trans-Golgi compartment [78] and with Lenhart et al. suggesting a role for receptor activity-modifying protein 3 (RAMP3) in the trafficking of GPER to the cell surface [79]. With the identification of an increasing number of GPCRs being expressed intracellularly [80, 81], particularly those GPCRs for membrane-permeable ligands (such as lipids and steroids) [82], as well as the recognized activities of internalized GPCRs [83], the cellular mechanisms and functional consequences of regulating GPER localization remain to be elucidated. With the recent characterization of differential subcellular activation of calcium stores by GPR55 depending on its site of activation in cardiomyocytes (plasmalemmal versus intracellular) [82], the subcellular site of GPER expression/activation may similarly play an important role in regulating its downstream signaling activity, particularly given the broad scope of GPER expression and function throughout the body [16, 69, 84–87].

Whereas the classical estrogen receptors (ER*α* and ER*β*) are traditionally thought to mediate primarily genomic responses [88], GPER has become recognized as an estrogen receptor that mediates rapid cellular signaling [89]. Nevertheless, there is also substantial evidence that ER*α* can mediate rapid signaling [90] and that GPER mediates transcriptional regulation [91]. Whereas ER*α* regulates transcription in part through direct binding to estrogen response elements in DNA [88], GPER presumably regulates transcription indirectly through kinase cascades [91]. While the transcriptional mechanisms of each receptor are distinct, there exist many possibilities for overlap and interactions of signaling activities (both synergistic and inhibitory) that remain largely undefined. The end results of GPER activation are also likely to be different in cells that also express ER*α* (or ER*β* for that matter) compared to cells that express only GPER. For example, the role of GPER in ER*α* ^+^ breast cancer cells that are “addicted” to ER-mediated signaling/gene expression for growth, and thus sensitive to antiestrogen/hormone therapy, may be very different compared to ER^−^/triple negative cells. The same may be true of endometrial cancers with ER*α* ^+^ type I tumors behaving very differently compared to ER^−^ type II tumors, particularly with respect to estrogen signaling. Furthermore, the effects of additional growth promoting factors/pathways may obscure or minimize the effects of estrogen through GPER, or ERs for that matter.

In this study, we demonstrated that an endometrial cell line representative of ER^−^ type II tumors maintains the ability to signal in response to estrogen via GPER. In Hec50 cells, estrogen signaling via GPER results in a metalloproteinase/EGFR-dependent activation of downstream kinase pathways, including PI3K and ERK, both important players in cancer cell survival and growth. Interestingly, the plasma membrane localization of PIP3 under unstimulated conditions suggests a level of constitutive PI3K activity in Hec50 cells. The ability of an EGFR inhibitor to reduce this further suggests that one or more members of the erbB family are involved through EGFR activation [92]. It is important to note that the effect of estrogen and G-1 might not be easily detectable by measuring total cellular pAkt levels, for example, by Western blot, due to the constitutive activation of PI3K. Translocation of the PH-RFP reporter however allows selective detection of GPER-mediated PI3K activation in the absence of global changes in the total cellular level of PI3K activity. Such location/compartment-specific signaling activity may play an important role in the overall effect of any activated pathway.

The pharmacopeia of estrogen receptor ligands has been developed in the absence of consideration for their interaction with GPER. For example, the development of SERMs and SERDs was largely based on the development of compounds with high binding affinity to ER*α* and the effects of these compounds on transcriptional regulation (through estrogen response elements) and overall tissue-specific activities (breast cancer cell growth versus uterine effects such as imbibition). Furthermore, the search for ER*α* versus ER*β* (and *vice versa*) selectivity [68, 93, 94] has also been carried without consideration of GPER function. In many cases, ligands or therapeutic agents were developed long before GPER was even identified, tamoxifen dating back to the 1970s. In many studies, these agents exhibit unexpected activities, often stimulating rapid cellular or physiological responses similar to estrogen without the expected inhibitory effects [95–100]. Although the disparate activities of SERMs have been attributed to tissue differences in the expression of ER coregulators [101], the agonistic effects of SERDs are perhaps less easily explained by such mechanisms. With previous reports of tamoxifen [28] and ICI182,780 [29] agonism through GPER, and now with our current demonstration of the activity of Raloxifene on GPER, the mechanisms of action of these compounds are greatly complicated. For example, in addition to ER*α*, what role does GPER play in hormone resistance in breast cancer [30, 31, 102] and the increased incidence of endometrial pathology and cancer in women taking tamoxifen [32]? The agonism of these compounds on GPER may well play an important role.

With the identification of ER*β* in 1996 [103], it soon became clear that selective ligands would be a powerful tool in the characterization of the functions of the individual estrogen receptors as well as being therapeutically promising. Because the ligand binding pockets of ER*α* and ER*β* are almost identical, achieving this goal has been challenging [104]. Today, the most widely used “selective” agonists for ER*α* and ER*β* are propylpyrazoletriol (PPT) and diarylpropionitrile (DPN), respectively [63, 64]. PPT displays ~400-fold binding selectivity for ER*α* over ER*β*, whereas DPN exhibits only ~70-fold selectivity for ER*β* over ER*α* [65–68]. In our current study, we found that PPT (at 100 nM, with a weak response at 10 nM, unpublished observation) also acts as an agonist for GPER. The three estrogen receptor selective compounds PPT, DPN, and G-1 have recently been used to evaluate the estrogen receptor involved in a number of physiological or cellular responses [105–107]. Prior to the identification of G-1, only PPT and DPN could be used to “distinguish” between ER*α* and ER*β*. A growing number of reports are concluding, for example, that both ER*α* and GPER mediate responses based on the activity of both PPT and G-1 [108, 109]; however, based on our current results, these responses *could* be mediated solely by GPER, if other measures of receptor involvement are not also employed. Clearly, PPT is a potent agonist of ER*α*, with enhanced selectivity versus GPER, but, without additional approaches (siRNA or selective antagonists such as G15 and G36), conclusions must be drawn with care and it is possible that many of the interpretations in the related literature (particularly using high PPT concentrations, e.g., ≥10 nM) should be reevaluated with these considerations in mind.

GPER stimulation has been demonstrated to increase cell proliferation in a broad array of cell lines, including some endometrial cancer cell lines, suggesting a potential importance in one or more aspects of carcinogenesis [15, 20, 24, 26, 27]. In fact, in a very recent study, knocking down GPER expression in Hec1A endometrial cancer cells (as well as Ishikawa H cells) resulted in a reduction of tumor growth in athymic mice [20]. This result is consistent with the many studies that suggest GPER expression correlates with poor survival or indicators of poor outcome in endometrial, ovarian, and other cancers [33–37]. In particular, GPER expression represents a mechanism by which ostensibly estrogen-unresponsive tumors (often stated as such based on the lack of ER*α* expression alone) can maintain estrogen responsiveness. The significance of this estrogen responsiveness will clearly depend on the type of cancer and the other mechanisms/mutations involved in a specific cancer. Although it has been amply demonstrated that, in breast cancer, antiestrogens targeting ER*α* are highly effective, the role of estrogen in gynecological cancers is thought to be of less importance due to the lack of clear efficacy of antiestrogens [110]. Nevertheless, the endometrium, like the breast, is highly estrogen responsive in terms of proliferation and elevated tumor estrogen levels have been reported not only in ER*α* ^+^ type I but also in ER*α* ^−^ type II endometrial tumors [111]. Thus, if GPER expression and function play an important role, particularly in ER*α* ^−^ type II endometrial cancers, then treatment with SERMs and SERDs, functioning as GPER agonists, would be highly contraindicated.

To examine the role of GPER in endometrial tumor growth, we sought to establish and investigate xenograft tumors in mice. We first examined whether the highly disparate levels of GPER expression in H cells and Hec50 cells observed in tissue culture were maintained as xenografts. Overall, xenograft tumors of each cell type displayed the expected histological properties with respect to tumor morphology and GPER expression with Hec50 cell tumors expressing substantially higher levels compared to H cell tumors. Importantly, primary xenografts displayed similar morphologies and GPER expression levels and patterns to those assessed directly from the patient samples. This suggests that the xenograft model may be a useful adjunct in which to test the therapeutic efficacy of GPER-selective compounds (particularly GPER-selective antagonists). Of particular relevance, in the clinical setting, we found that low expression of GPER was associated with the only observed response to tamoxifen; and since this patient was strongly ER^+^/PR^+^, we surmise this effect may have been mediated by classical steroid receptor pathways [112].

To determine if estrogen itself and GPER in particular contributes to tumor growth of ER*α* ^−^ Hec50 cells, we established subcutaneous Hec50 tumors in ovariectomized athymic mice. Tumors remained small in untreated mice but were about 10-fold larger in mice treated with slow release estrogen pellets, indicating that the lack of ER*α* did not prevent responsiveness to estrogen. Interestingly, we were not able to demonstrate estrogen-stimulated growth in monolayer culture of Hec50 cells, suggesting estrogen-responsiveness may be a result of the tumor environment (unpublished results), highlighting the importance of tumorigenesis studies *in vivo*. To test whether GPER can mediate this estrogen responsiveness, we treated mice with slow release pellets containing G-1. These tumors were about half the size of the estrogen-treated mice, suggesting that GPER could mediate the effects of estrogen. The reduced tumor size of G-1-treated mice compared to estrogen-treated mice could be due to differential sensitivity to the two ligands (e.g., EC_50_ values) or differential distribution, metabolism, or excretion of the two compounds. Finally, to investigate more directly the role of GPER in the estrogen responsiveness, we treated mice with both estrogen and the GPER-selective antagonist G36. The tumors in these mice were restored to the size of the tumors in the sham-treated mice. These results indicate not only that the observed estrogen dependence of Hec50 cell tumors is due to GPER but also that pharmacological inhibition of GPER could represent an important new therapy for women, particularly those with aggressive type II endometrial cancer.

## 5. Conclusions

In this paper, we have demonstrated that GPER plays an important role in the estrogen-mediated signaling of a representative type II endometrial cancer cell line. In addition, we demonstrate for the first time that the SERM Raloxifene is an agonist for GPER, with potentially important clinical ramifications for its FDA-approved chronic use for osteoporosis. The ability of the widely used “ER*α*-selective” agonist PPT to activate GPER suggests that a large body of literature may have to be more carefully interpreted with respect to defining the roles of individual estrogen receptors. Finally, we have demonstrated that GPER-selective antagonists may represent important new therapeutic agents for endometrial and other cancers pathologically defined as estrogen unresponsive due to their lack of ER*α* expression.
